# Improving Control of Gene Therapy-Based Neurotrophin Delivery for Inner Ear Applications

**DOI:** 10.3389/fbioe.2022.892969

**Published:** 2022-06-03

**Authors:** Madeleine St. Peter, Douglas E. Brough, Anna Lawrence, Jennifer Nelson-Brantley, Peixin Huang, Jennifer Harre, Athanasia Warnecke, Hinrich Staecker

**Affiliations:** ^1^ University of Kansas School of Medicine, Kansas City, KS, United States; ^2^ Precigen Inc., Gaithersburg, MD, United States; ^3^ Department of Otolaryngology, University of Kansas School of Medicine, Kansas City, KS, United States; ^4^ Department of Otolaryngology, Hannover Medical School, Hannover, Germany

**Keywords:** brain-derived neurotrophic factor, neurotrophin 3, spiral ganglion neurons, herpes latency promoter, ototoxicity

## Abstract

**Background:** Survival and integrity of the spiral ganglion is vital for hearing in background noise and for optimal functioning of cochlear implants. Numerous studies have demonstrated that supplementation of supraphysiologic levels of the neurotrophins BDNF and NT-3 by pumps or gene therapy strategies supports spiral ganglion survival. The endogenous physiological levels of growth factors within the inner ear, although difficult to determine, are likely extremely low within the normal inner ear. Thus, novel approaches for the long-term low-level delivery of neurotrophins may be advantageous.

**Objectives:** This study aimed to evaluate the long-term effects of gene therapy-based low-level neurotrophin supplementation on spiral ganglion survival. Using an adenovirus serotype 28-derived adenovector delivery system, the herpes latency promoter, a weak, long expressing promoter system, has been used to deliver the BDNF or NTF3 genes to the inner ear after neomycin-induced ototoxic injury in mice.

**Results:** Treatment of the adult mouse inner ear with neomycin resulted in acute and chronic changes in endogenous neurotrophic factor gene expression and led to a degeneration of spiral ganglion cells. Increased survival of spiral ganglion cells after adenoviral delivery of BDNF or NTF3 to the inner ear was observed. Expression of BDNF and NT-3 could be demonstrated in the damaged organ of Corti after gene delivery. Hearing loss due to overexpression of neurotrophins in the normal hearing ear was avoided when using this novel vector–promoter combination.

**Conclusion:** Combining supporting cell-specific gene delivery via the adenovirus serotype 28 vector with a low-strength long expressing promoter potentially can provide long-term neurotrophin delivery to the damaged inner ear.

## Introduction

The spiral ganglion plays a vital role in hearing, ranging from transfer of the auditory signal to initial encoding that allows hearing in background noise (Rusznák & Szucs, 2009; Green et al., 2012). Loss of spiral ganglion neurons or loss of synaptic contacts between the spiral ganglion and the inner hair cells can result in decreased speech discrimination, poor hearing in background noise, and, in the cases of cochlear implantation, poor post-implant outcomes (Seyyedi, Viana, & Nadol, 2014). Loss of trophic factor production is thought to be the main reason for the degeneration of spiral ganglion cells (Bailey & Green, 2014). Indeed, animal models have demonstrated that loss of trophic support after damage of the organ of Corti results in progressive degeneration of the neuronal population within the spiral ganglion (Atkinson et al., 2012; Budenz et al., 2015; H; Staecker, Jolly, & Garnham, 2010). Supplementation of neurotrophic factors including brain-derived neurotrophic factor (BDNF), neurotrophin 3 (NT-3), and glial cell-derived neurotrophic factor (GDNF) has been proposed for improving spiral ganglion survival and enhancing cochlear implant outcomes (Bailey & Green, 2014; Pfingst et al., 2017a; Dabdoub & Nishimura, 2017; Konerding et al., 2017; Plontke et al., 2017; Senn et al., 2017). Although many different growth factors are expressed within the inner ear, research on neurotrophic factor supplementation therapy for cochlear implantation has predominantly focused on the neurotrophins BDNF and NT-3. In animal models, the additional delivery of various growth factors to the cochlea has resulted in increased preservation of neurons after deafness, re-growth of peripheral processes, and reduction of the threshold of electrical stimulation, suggesting that combining delivery of neurotrophins or neurotrophin mimetics may improve cochlear implant function (Pfingst et al., 2015). This has been corroborated by follow-up studies demonstrating a positive impact of neurotrophin supplementation on cochlear implant function (Pfingst et al., 2017; Leake et al., 2019). Neurotrophin therapy may have additional indications including mitigating noise-induced synaptopathy (Wan et al., 2014). Despite promising results from experimental research over the past decades, no validated pharmacological treatment to support neuronal health in the inner ear has been derived for clinical application yet. One reason could be the lack of understanding of neurotrophin and growth factor biology in the adult auditory system. This is equally true for other organs and brain regions where the cellular localization of the neurotrophin is difficult to detect due to the very low levels of the endogenous protein (Dieni et al., 2012). The role and distribution of neurotrophins especially within the inner ear is incompletely understood, due to the difficulty in accessing the sensory region. For example, denaturation of proteins during the decalcification procedure of the temporal bone that is required for immunohistochemistry may hamper detection of neurotrophin levels. Detection of neurotrophins in the inner ear in an embryonic or newborn state is much less demanding. However, these data may not transfer to the adult animal due to shifts in neurotrophin expression during maturation. Expression of BDNF in adult mice is limited to the pillar cells in the cochlea and to the vestibular supporting cells (Singer et al., 2014). During adulthood, NT-3 is localized to the inner hair cells and vestibular supporting cells (Mitsuru Sugawara et al., 2007). *In situ* hybridization studies also suggest the presence of BDNF and NTF3 gradients, basal (BDNF) and apical (NTF3), in the spiral ganglion of adult rats (Schimmang et al., 2003). The interaction between hair cells, supporting cells, and neurons may additionally play an important role in the growth factor production and support of neuronal integrity (M Sugawara et al., 2005). Analysis of neurotrophin signaling pathways suggest that changes in neurotrophin signaling should result in a broad range of physiologic changes in the cochlea beyond neuronal survival (Ramekers et al., 2012). Thus, it is important to focus on physiological levels of delivery to maintain or to restore endogenous neurotrophin homeostasis.

Delivery studies in animal models have used pumps, engineered cell lines, and gene therapy strategies to improve spiral ganglion survival after damage to the organ of Corti (Dabdoub & Nishimura, 2017). Among these, gene therapy strategies have the advantage of potentially providing supplementation of the growth factor for years, which would be required for human translation. Adeno-associated vectors (AAV) are currently widely applied for long-term gene delivery in the inner ear, providing long-term expression by the target tissue (Lustig & Akil, 2012). Interestingly, recent studies that investigated delivery of NTF3 in a normal hearing ear demonstrated that overexpression of growth factors could result in hearing loss and abnormal neuritic growth (Lee et al., 2016a). Human cochlear implant patients frequently have residual hearing in low frequencies, so translation of neurotrophin therapy into these populations must consider these potential side effects.

We have previously demonstrated the ability of the rare serotype adenoviral vector, Ad28, to target supporting cells (H. Staecker et al., 2007; Hinrich Staecker et al., 2014). The herpes simplex virus (HSV) latency (lat) promoter has been widely studied and has been shown to drive expression at a low level over long time periods (Chattopadhyay et al., 2005).

We, thus, evaluated the combination of Ad28 with the HSV lat promoter to drive a low-level but long-term expression of BDNF or NT-3 in the inner ear. The effects of promoter choice were evaluated *in vitro* and in normal hearing animals *in vivo*. Finally, we evaluated the effects of lat promoter-driven neurotrophin expression in an animal ototoxicity model. The combination of Ad28 vector properties with the use of a low strength stable promoter yields a novel way to direct and control the expression of neurotrophins in the inner ear.

## Materials and Methods

Animals: All procedures were approved by the KUMC IACUC 2018-2442. For all experiments, 1-month-old C57Bl/6 mice were used.

Vector construction and storage: The production system for the Ad28 adenovectors provides generation of an E1, E3 deleted replication-deficient adenovector with purified stocks at 5 × 10^11^ to 2 × 10^12^ total particles (particle unit, pu) per mL with a total particle to active particle (fluorescent focus unit, ffu) ratio ranging from 3 to 10 pu/ffu. Total particles (pu) were determined by a spectrophotometric assay. Adenovector lots were purified, aliquoted, and stored at −80°C. Individual aliquots were used for each experiment to prevent loss of activity associated with freeze thaw cycles. Vectors produced included Ad28 with the human cytomegalovirus (hCMV) promoter CMV promoter driving green fluorescent protein (GFP), BDNF, and NTF3 genes and Ad28 with HSV latency promoter (lat) driving BDNF and NTF3. Prior to *in vitro* or *in vivo* use, vectors were diluted to equivalent maximum concentrated titers. A total of five animals were used for each vector condition.

### *In Vitro* Assessment of Neurotrophin Production

Transfection**:** Whole inner ears were dissected from C57Bl/6 mice and utricles were removed. The utricles were placed on a 0.45-µm Millicell-HA culture insert (cat# PIHA01250) in a 24-well plate with media (DMEM, N1 supplement, penicillin, neomycin 10^−3^). This process was repeated for each subsequent dissection. The tissues were incubated for 48 h at 37°C/5% CO_2_ to induce injury. The vector was added to fresh media (1 µl vector/400 µl medium) into a new 24-well plate and returned to the incubator. The medium was collected and replaced every 72 h ending on the 27th day of collection. The media were then placed in −20°C freezer until further analysis. Analysis was performed in triplicate with five utricles per membrane.

Enzyme-linked immunosorbent assay (ELISA): ELISAs specific for BDNF and NT-3 were used to detect expression levels in the supernatants. Explant supernatant was thawed, and reactivity was tested for the neurotrophins (BDNF: PicoKine^™^ ELISA Kit, Boster Biological Technology #EK0307/9; NT-3: mouse Neurotrophin-3 ELISA Kit, Abcam, ab213882).

A subset of neomycin medium collected from neomycin-treated explants which were incubated with Ad28.lat.bdnf were microdialyzed using Pall Nanosep^®^ Filters (Pall Corp, Westborough, MA United States) per manufacturer’s instructions and subsequently assayed for BDNF content as outlined above.

### *In Vivo* Delivery of Adenovectors to the Inner Ear

Surgical procedure: Mice were anesthetized with an intraperitoneally administered mixture of ketamine (100 mg/kg), xylazine (5 mg/kg), and acepromazine (2 mg/kg). A dorsal postauricular incision was made and the bulla exposed. Using an 18# needle, a hole was drilled exposing the middle ear space medial to the tympanic ring. The round window niche was identified and the bone overhanging the niche was scraped away revealing the round window membrane. A measure of 1 μl of sterile neomycin was injected into the round window that was then sealed (H. Staecker et al., 1998). Animals were allowed to recover for 1 week and hearing loss was then confirmed by auditory brain stem response (ABR) testing. For vector delivery, the animals were anesthetized and the posterior semicircular canal exposed as previously described (Pfannenstiel et al., 2020). Vector injections consisted of 10^8^ pu of vector in 1 µl of volume (delivered using a Hamilton micro syringe with 0.1 µl graduations). For all *in vivo* vector delivery experiments, animals survived for 1 month post vector delivery.

ABR Measurement: ABR thresholds were recorded using the Intelligent Hearing Systems Smart EP program (IHS, Miami, FL, United States). Animals were anesthetized as described above and kept warm on a heating pad (37°C). Needle electrodes were placed on the vertex (+), behind the left ear (−), and behind the opposite ear (ground). Tone bursts were presented at 4, 8, 16, and 32 kHz, with duration of 500 μs using a high-frequency transducer. Recording was carried out using a total gain equal to 100 K and using 100-Hz and 15-kHz settings for the high- and low-pass filters. A minimum of 128 sweeps were presented at 90 dB SPL. The SPL was decreased in 10 dB steps. Near the threshold level, 5 dB SPL steps using up to 1,024 presentations were carried out at each frequency. Threshold was defined as the SPL at which at least one of the waves could be identified in two or more repetitions of the recording.

Immunohistochemistry: Mice were anesthetized with intraperitoneal applications of phenobarbital (585 mg/kg) and phenytoin sodium (75 mg/kg) (Beuthanasia^®^-D Special, Schering-Plough Animal Health Corp., Union, NJ, Canada) and killed via intracardiac perfusion with 4% paraformaldehyde in PBS. The temporal bones were removed, the stapes extracted, and the round window was opened. The temporal bones were postfixed overnight in 4% paraformaldehyde in PBS at 4°C. After rinsing in PBS three times for 30 min, the temporal bones were decalcified in 10% ETDA (ethylene diamine tetracetic acid) for 48 h. The temporal bones were rinsed in PBS, dehydrated, and embedded in paraffin. Ten-µm sections were cut in parallel to the modiolus, mounted on Fisherbrand^®^ Superfrost^®^/Plus Microscope Slides (Fisher Scientific, Pittsburgh, PA, United States) and dried overnight. Samples were deparaffinized and rehydrated in PBS two times for 5 min, then three times in 0.2% Triton X-100 in PBS for 5 min and, finally, in blocking solution 0.2% Triton X-100 in PBS with 10% fetal bovine serum for 30 min at room temperature. After blocking, specimens were treated with antibodies listed in Table 1 and diluted to the listed concentration with blocking solution. The tissue was incubated for 48 h at 4°C in a humid chamber. After three rinses in 0.2% Triton X-100 in PBS, immunohistochemical detection was carried out with an Alexa Fluor 555 Donkey anti-rabbit (1:1,000; Abcam). The secondary antibody was incubated for 6 h at room temperature in a humid chamber. The slides were rinsed in 0.2% Triton X-100 in PBS three times for 5 min and finally cover slipped with ProLong^®^ Gold antifade reagent (Invitrogen™ Molecular Probes, Eugene, OR, United States). Neuronal survival was quantified in the basal and apical turns of three specimens from each treatment group. Mid-modiolar sections were imaged and high-power images of the spiral ganglion at the basal and most apical turn imported into NIS elements (Nikon Inc., Melville, NY). Neurons staining with neurofilament that had a normal-appearing nucleus were counted in a square region in the center of the ganglion. The NIS software was used to measure the area counted, allowing calculation of a neuronal density. For whole mount neuronal imaging, temporal bones were washed in PBS for 20 min after decalcification. The otic capsule and stria were removed. Segments of the spiral ganglion and adjacent organ of Corti were removed and placed in PBS. The samples were placed in blocking solution for 30 min and then incubated with anti-neurofilament (Abcam) overnight. The samples were then washed in PBS three times for 1 h followed by secondary antibody (Alexa Fluor 555 Donkey anti-rabbit (1:1,000; Abcam)) for 12 h. Samples were again washed in PBS three times for 30 min and then mounted on slides with ProLong^®^ Gold antifade reagent.

**TABLE 1:** List of primary antibodies used for immunohistochemistry.

Antibody	Company	Product #	Concentration (mg/ml)	Dilution/concentration (μg/ml)	Source	Type
Anti-NF	Abcam	ab8135	1.0	1:300 (3.33)	Rabbit	Polyclonal
Anti-TrkB	Alomone Labs	ANT-019-AG	1	1:50 (20)	Rabbit	Polyclonal
Anti-TrkC	Alomone Labs	ANT-020	0.8	1:50 (16)	Rabbit	Polyclonal
Anti-BDNF	Alomone Labs	ANT-010	0.72	1:50 (14.4)	Rabbit	Polyclonal
Anti-NT3	Alomone Labs	ANT-003	0.3	1:200 (1.5)	Rabbit	Polyclonal

RNA isolation and mRNA profiling: Total RNA from whole mouse cochlea was extracted using TRIzol reagent (ThermoFisher, cat #15596018) and purified with phase-lock heavy gel (Qiagen, cat# WMS-2302830). Three animals were used for each condition and time point. RNA concentration and integrity was assessed using the Agilent 2100 Bioanalyzer (Agilent, Santa Clara, CA, United States). Samples with an RNA integrity number (RIN) greater than 5.5 were included in further processing. Using the RT^2^ First Strand Kit (Qiagen, cat#330401), 0.5 µg of RNA was converted to cDNA. qRT-PCR was then performed via the RT^2^ mouse neurotrophins and receptors SYBR Green PCR array (Qiagen, cat#PAMM-031Z). Cycling conditions were programmed as specified in the supplier handbook. All gene expression values were normalized against the average C_t_ of the five mouse reference genes: Gapdh, Actb, Gusb, hsp90, and B2M. The relative expression for each gene was calculated using the ΔΔC_t_ method based on three replicate runs. Significant genes were selected according to cut-off values *p* < 0.05 and fold-change >2 or <0.5 as determined by the manufacturer provided software.

Statistics: ANOVA and ANOVA for repeated measures were carried out using GraphPad Prism V 8. A value of *p* < 0.05 was considered significant. Error bars represent standard deviation.

## Results

### The Herpes Latency Promoter Drives Low Levels of Neurotrophin Expression *In Vitro*

To evaluate the effect of a latency promoter on neurotrophin gene expression, adult mouse utricle explants were harvested and treated for 48 h with neomycin (10^−3^ M). The explants were then maintained *in vitro* for 27 days. Control explants were placed in defined medium after aminoglycoside challenge. Experimental groups consisted of neomycin-treated utricles exposed for 24 h to Ad28 vector carrying either the BDNF gene or the NTF3 gene driven by the herpes latency promoter or Ad28 vector carrying the BDNF gene or NTF3 driven by the hCMV promoter. Each explant was maintained in its own well, and medium was collected and exchanged for fresh defined medium every 72 h. At the end of the experiment, the medium for each time point from each explant was evaluated for the expression of either BDNF or NT-3 using commercially available ELISA kits.

**FIGURE 1:**
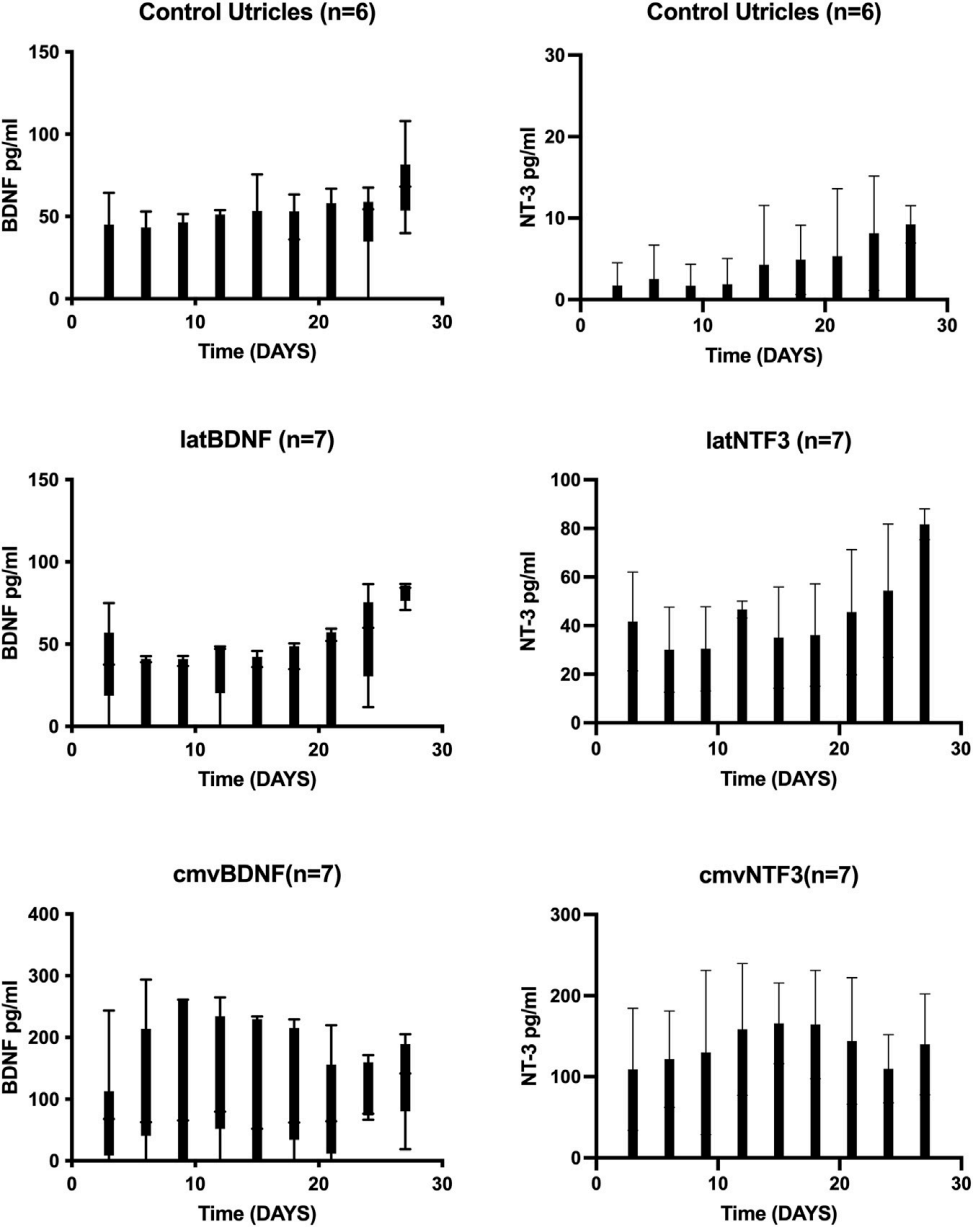
Expression of BDNF and NT-3 in neomycin-treated utricle cultures over time. Adult mouse utricles were treated with neomycin for 48 h and maintained *in vitro* for 1 month. Controls were maintained in defined medium, and experimental groups were treated with an Ad28 vector delivering the BDNF or NTF3 genes driven either with the herpes latency promoter or the human CMV promoter. Medium was exchanged every 72 h, and BDNF or NT-3 levels were determined by ELISA. Error bars represent standard deviation. Controls and the lat promoter groups showed a low production of BDNF at the limits of the ELISA sensitivity. Utricles treated with the CMV-driven BDNF showed steady growth of BDNF expression over the first 10 days *in vitro* that was maintained at high levels for the duration of the experiment. NT-3 was barely detectable in control utricle explants. NTF3 driven by the lat promoter produced a low level of NT-3, whereas use of a CMV promoter resulted in higher expression levels of NT-3.

Utricle explants treated with neomycin showed production of a low level of BDNF and barely detectable levels of NT-3 (Figure 1). Delivery of Ad28 with the lat promoter driving either BDNF or NTF3 resulted in low levels of neurotrophin production over the next 27 days. Levels of BDNF and NT-3 ranged from 10 to 70 pg per ml (pg/ml) produced every 72 h. Comparing untreated neomycin-exposed utricles to the lat promoter-driven neurotrophin-treated utricles demonstrated a measurable increase of NT-3 production, whereas production of BDNF-treated explants did not seem to be higher. For comparison, we also used a strong promoter (hCMV) to drive neurotrophin expression in utricle explants. Explants treated with either Ad28 carrying the hCMV promoter driving the BDNF gene or Ad28 carrying the hCMV-driven NTF3 resulted in significantly higher levels of neurotrophin production. In both experimental conditions, neurotrophin production ranged around 100 pg/ml per 72 h over the length of the experiment (Figure 1).

**FIGURE 2:**
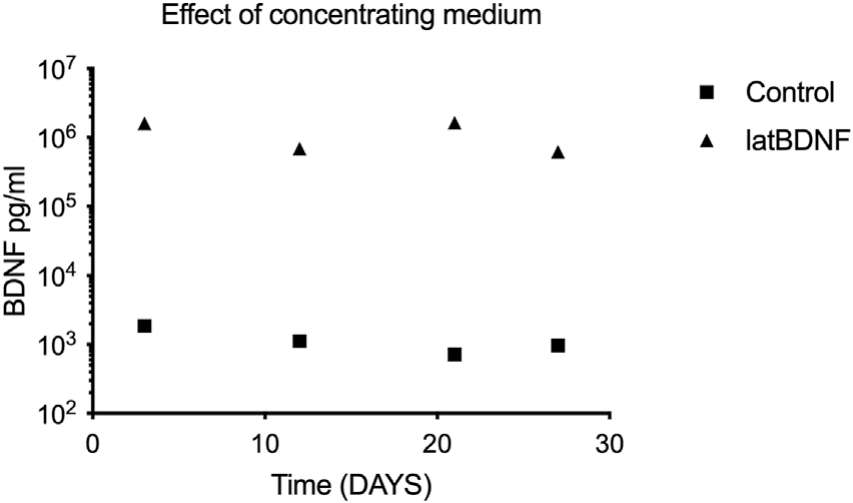
To evaluate differences between control utricle production of BDNF and cultures treated with Ad28.lat.BDNF, a long-term culture experiment was carried out. Medium from the cultures was collected at four time points and concentrated *via* microdialysis. ELISA for BDNF showed that controls expressed a consistent low level of BDNF, whereas the Ad28.lat.BDNF-treated cultures showed higher levels of BDNF expression.

Since no significant differences in BDNF production were seen in the control (untreated) and the lat BDNF-treated explants, a second cohort of control and Ad28.lat.BDNF-treated explants were maintained *in vitro* for 1 month. At days 3, 12, 21, and 27 *in vitro*, medium was collected, pooled, and microdialyzed. When comparing the Ad28.lat.BDNF-treated medium to the medium collected from neomycin-only-treated utricles, a clearly higher level of BDNF production could be shown in the treated utricles (Figure 2). This likely suggests that the assay was not sensitive enough to differentiate additional low-level BDNF production induced by the vector over endogenous BDNF production from the utricular supporting cells. However, we can conclude that the vector system does induce a measurable low level of BDNF production.

### The Ad28 Vector Can Deliver Genes to the Damaged Organ of Corti

**FIGURE 3:**
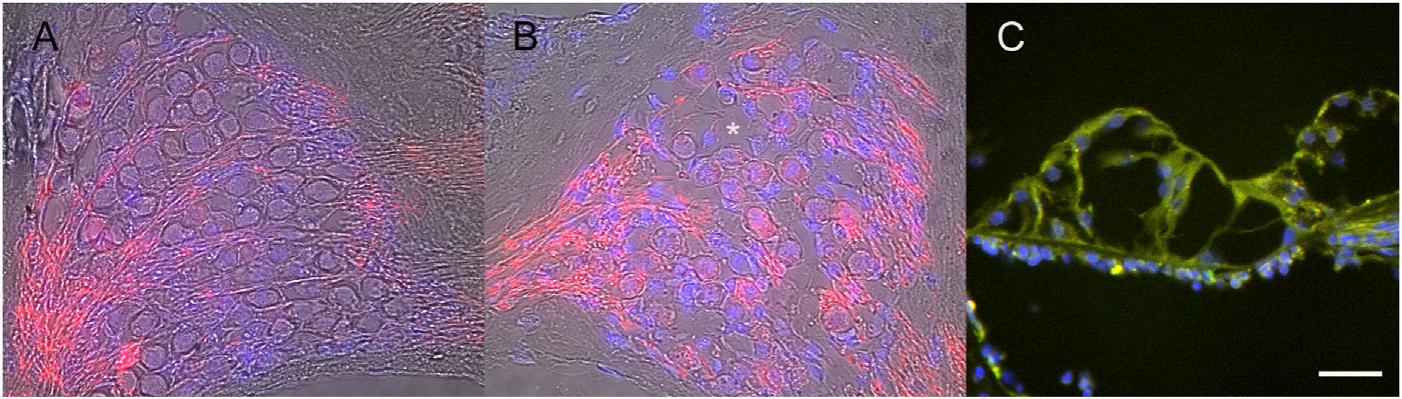
Use of an Ad28 vector (Ad28.hcmv.gfp) did not decrease neuronal survival in normal hearing inner ears **(A)** and conversely did not support survival on spiral ganglion cells after ototoxicity **(B)**. Asterisk in B marks empty vacuole due to lost spiral ganglion neuron. There is expression of GFP in the neomycin-treated organ of Corti after delivery of Ad28.hcmv.gfp **(C)**. Scale bar = 20 µm **(A,B)**, 10 µm **(C)**.

Ad28 vectors are based on a rare serotype of adenovirus that does not have significant levels of immune response against the virus in human populations (Hamilton et al., 2008). To look at the distribution and potential effects of the vector itself, we delivered 1 × 10^8^ particles of Ad28 carrying the green fluorescent protein (GFP) gene driven by the CMV promoter (Ad28.hcmv.gfp) to both normal hearing and neomycin-treated mice. There is no effect of vector delivery on the integrity of the spiral ganglion in normal hearing mice (Figure 3A). Treatment of mice with Ad28.hcmv.gfp 3 days after injection of neomycin to the inner ear did not appear to improve spiral ganglion survival (Figure 3B). Evaluations of the organ of Corti at 1 month post neomycin and Ad28.hcmv.gfp delivery shows loss of hair cells and GFP-positive cells within the damaged organ of Corti (Figure 3C), suggesting that use of this vector capsid results in the transgene being expressed in the appropriate target cells (surviving supporting cells in the organ of Corti).

### Overexpression of Neurotrophins Can Induce Hearing Loss in Normal Hearing Animals

**FIGURE 4:**
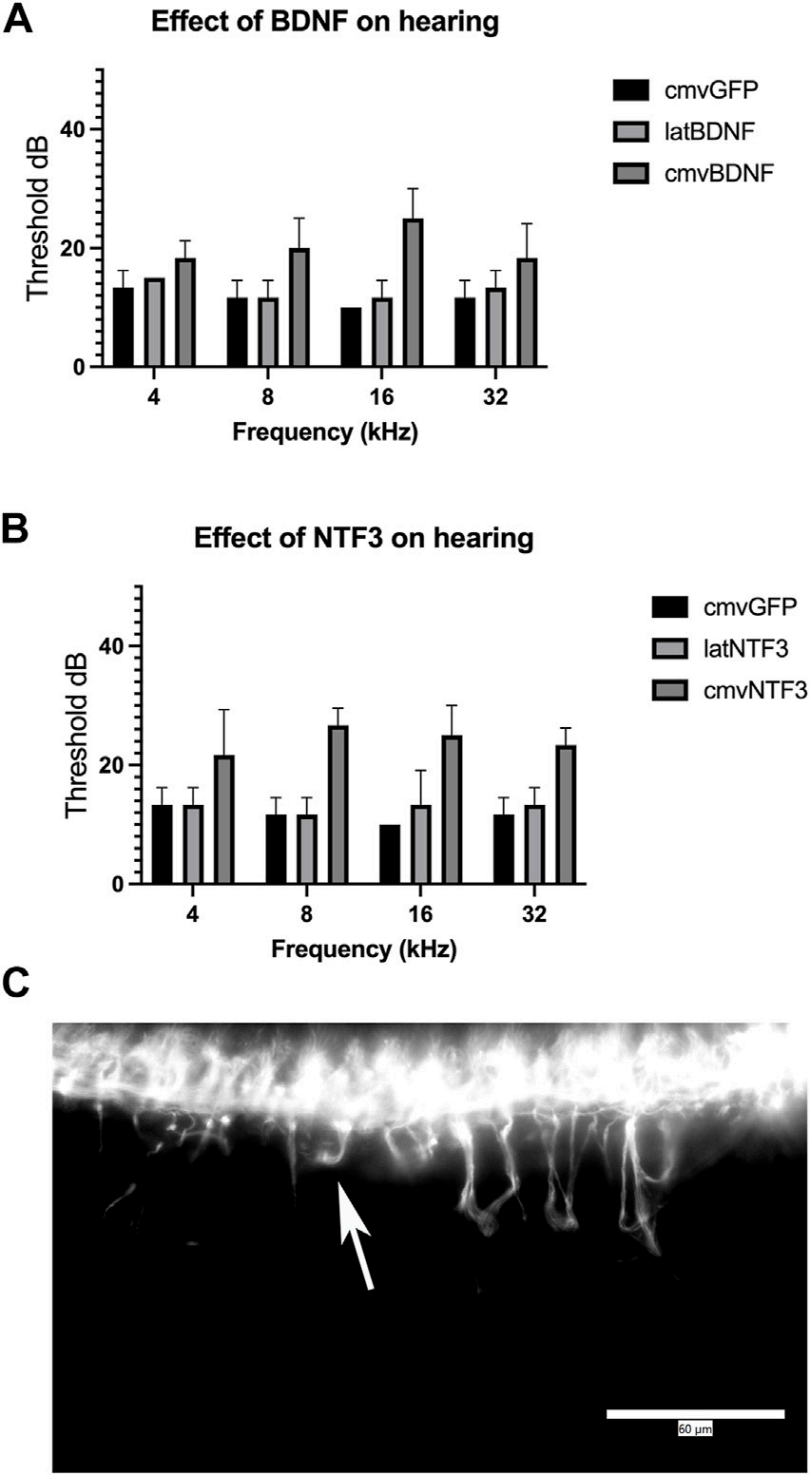
Effect of neurotrophin expression in the normal hearing ear. Expression of BDNF in the cochlea appeared to induce a mild change in hearing when expressed at high levels using the CMV promoter **(A)**. This was not significant compared with expression driven by the lat promoter or overexpression of GFP driven by the CMV promoter. Overexpression of NTF3 at high levels caused significant elevation of thresholds at 8 k, 16 k, and 32 kHz, compared with lat-driven NTF3 or control inner ears **(B)**. Evaluation of spiral ganglion neurons by immunofluorescence demonstrated abnormal migration of spiral ganglion neurites after delivering the NTF3 gene with a strong promoter [**(C)**, Arrow]. Error bars represent standard deviation (*n* = 5). Scale bar = 60 µm.

One-month-old mice were treated with matched doses (5 × 10^8^ pu) of Ad28.cmv.gfp, Ad28.lat.bdnf, Ad28.lat.ntf3, Ad28.cmv.bdnf, or Ad28.cmv.ntf3. This results in delivery of a low- and a high dose of either BDNF or NT-3 to the normal hearing inner ear. One month post vector delivery, hearing was checked by ABR. Ad28.hcmv.gfp did not affect hearing measured 1 month post-delivery via a posterior canal canalostomy. Animals treated with Ad28.hcmv.gfp or Ad28.lat.bdnf showed similar thresholds. Treatment with Ad28.hcmv.bdnf resulted in slight elevation of hearing thresholds in the high frequencies. This was, however, not statistically significant (*p* = 0.469 at 32 kHz) (Figure 4A). Ad28.lat.ntf3-treated animals showed similar thresholds as animals treated with Ad28.hcmv.gfp. Delivery of Ad28.cmv.ntf3 resulted in statistically significant threshold elevations at 8, 16, and 32 thousand Hz (*p* = 0.003, 0.0035, and 0.012 at 32 kHz) (Figure 4B). This suggests that NT-3 delivered at high doses can result in hearing loss in normal hearing animals. Whole mount imaging of spiral ganglion neurites demonstrated that there was abnormal growth of neurites in the animals treated with Ad28.cmv.ntf3 (Figure 4C).

### Aminoglycoside Ototoxicity Reduces Cochlear Neurotrophin Gene Expression

One-month-old mice were treated with an injection of concentrated neomycin into the posterior semicircular canal. One week post neomycin treatment, the mice were anesthetized and hearing was checked by ABR. To determine changes in growth factor mRNA expression over time, mice with profound hearing loss were then divided into two groups. Mice in Group 1 were killed, and their cochlear mRNA was extracted. Mice in Group 2 were allowed to survive further 3 weeks and then killed, and their mRNA was extracted from the neomycin-treated ear. An age-matched cohort of normal hearing mice was additionally killed and underwent extraction of cochlear mRNA to serve as a control. Levels of neurotrophin mRNA were then quantified using a semiquantitative RT-PCR. At 7 days post neomycin treatment, there was an abrupt downregulation in BDNF and NTF3 mRNAs as well as downregulation of the TrkB and TrkC receptors compared with untreated controls (Table 2). BDNF mRNA initially decreased by –4.1 ± 0.2 fold (*p* < 0.015). By 1 month post neomycin, BDNF mRNA showed a mild relative downregulation (−1.6 ± 0.05 fold, *p* < 0.05) compared with controls. NTF3 mRNA showed an initial −2.7 ± 0.2 fold (*p* < 0.014) drop relative to controls which normalized at 1 month post neomycin treatment (−1 ± 0.5 fold *p* = 0.154). Expression of TrkB receptor mRNA initially decreased by –4.5 ± 0.2 fold (*p* < 0.05) and remained significantly downregulated at 1 month post neomycin treatment (−2.8 ± 0.09 fold, *p* < 0.01). TrkC mRNA initially declined by −3.73 ± 0.4 fold (*p* < 0.05) and at 1 month post neomycin showed a −4.28 ± 0.1 fold (*p* < 0.01) downregulation compared with normal hearing controls (Table 1). The PCR array used in the analysis also queries other factors that have been explored in the ear. Ciliary-derived neurotrophic factor (CNTF), glial cell line-derived neurotrophic factor (GDNF), basic fibroblast growth factor (bFGF), and transforming growth factor *β* (TGFβ) showed significant downregulation acutely with relatively lower rates of downregulation at 1 month post neomycin treatment (Table 2).

**TABLE 2:** qRT-PCR evaluation of growth factor expression 1 week and 1 month after neomycin treatment. Results show expression relative to non-neomycin-treated controls.

Gene name	Description	1 week post-neo	1 month post-neo
Artn	Artemin	−1.1	1.3
Bdnf	Brain-derived neurotrophic factor	−4.1	−1.6
Cntf	Ciliary neurotrophic factor	−4.1	−1.5
Cntfr	Ciliary neurotrophic factor receptor	−7.9	−1.8
Fgf2	Fibroblast growth factor 2	−2.3	−2.1
Fgf9	Fibroblast growth factor 9	−6.9	−1.9
Fgfr1	Fibroblast growth factor receptor 1	−3.6	−1.6
Frs2	Fibroblast growth factor receptor substrate 2	−3.5	−1.7
Frs3	Fibroblast growth factor receptor substrate 3	−5.1	−2.3
Gdnf	Glial cell line-derived neurotrophic factor	−1.9	1.7
Gfra1	Glial cell line-derived neurotrophic factor family receptor alpha 1	−2.8	−1
Gfra2	Glial cell line-derived neurotrophic factor family receptor alpha 2	−2.4	1.3
Gfra3	Glial cell line-derived neurotrophic factor family receptor alpha 3	−1.2	−1.9
Ngf	Nerve growth factor	−1.4	−1.2
Ngfr	Nerve growth factor receptor (TNFR superfamily, member 16)	–1.5	–2.3
Ngfrap1	Nerve growth factor receptor (TNFRSF16)-associated protein 1	–2.7	–1.4
Nrg1	Neuregulin 1	–2.3	−1.4
Nrg4	Neuregulin 4	−1.8	−2
Ntf3	Neurotrophin 3	−2.7	−1
Ntf5	Neurotrophin 5	−3.3	−1.5
TrkA	Neurotrophic tyrosine kinase, receptor, type 1	−4.7	−2.1
TrkB	Neurotrophic tyrosine kinase, receptor, type 2	−4.5	−2.8
TrkC	Neurotrophic tyrosine kinase, receptor, type 3	−3.73	−4.3
Pspn	Persephin	−6.2	−1.5
Tgfa	Transforming growth factor alpha	−3	−1
Tgfb1	Transforming growth factor, beta 1	−2.5	1.2
Tgfb1i1	Transforming growth factor beta 1 induced transcript 1	−2.3	−1.3

### Lat Promoter-Driven Neurotrophin Expression Induces Spiral Ganglion Survival After Ototoxin Exposure

**FIGURE 5:**
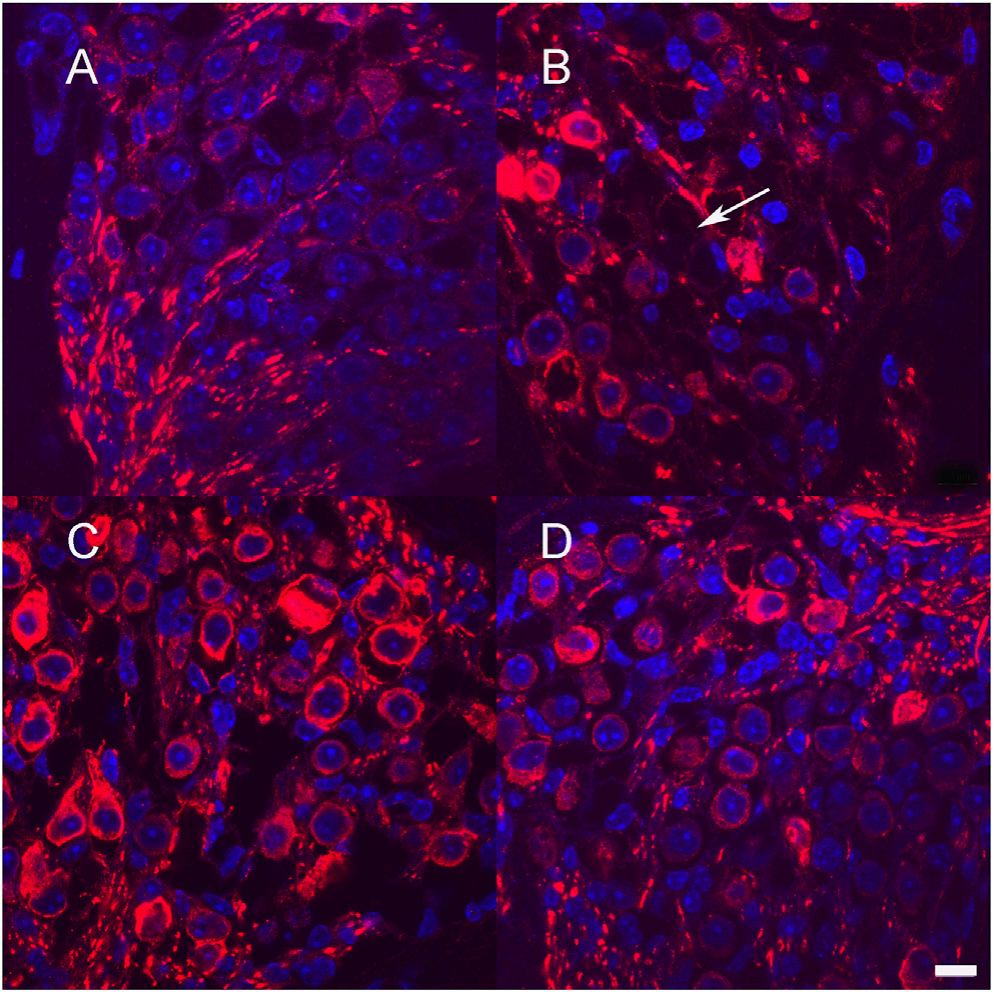
Neuronal survival after low-level neurotrophin gene therapy using the lat promoter **(A)**. Section through Rosenthal’s canal in the basal turn of an adult hearing mouse shows spiral ganglion neurons immunostained for neurofilament (red). Cell nuclei are stained in blue using DAPI injection of neomycin into the round window that resulted in loss of greater than 50% of spiral ganglion cells **(B)** Example of missing neurons are shown with arrow **(B)** Neomycin treatment followed by delivery of Ad28.lat.bdnf into the posterior semicircular canal resulted in improved survival of spiral ganglion cells **(C)**. Animals treated with Ad28.lat.ntf3 after neomycin treatment resulted in optimal neuronal survival and morphology similar to controls **(D)**. Scale bar = 10 µm.

**FIGURE 6:**
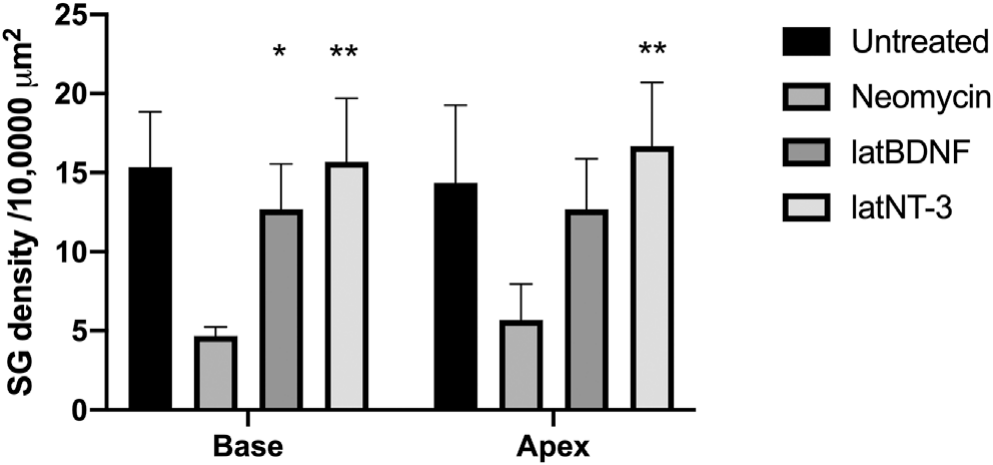
Neuronal survival after neomycin treatment and after neomycin treatment followed by delivery of Ad28.lat.bdnf or Ad28. lat.ntf3. Delivery of BDNF resulted in statistically significant improvement of spiral ganglion survival in the basal turn. NT-3 delivery significantly improved spiral ganglion survival in both the basal and apical turns. Error bars represent standard deviation (*n* = 3).

One-month-old mice were treated with neomycin injection into the round window. One week post treatment, animals were evaluated by ABR to document complete loss of hearing. Control neomycin-only-treated animals were then injected with 1 μl of artificial perilymph delivered into the posterior semicircular canal and then allowed to survive for 1 month. Two further groups of animals that had been treated with neomycin were then additionally treated with either Ad28.lat.bdnf or Ad28.lat.ntf3 delivered into the posterior semicircular canal and then allowed to recover for 1 month. Age-matched, untreated mice demonstrated a healthy spiral ganglion population with normal distribution of neurofilament with an average neuronal density of 15.3 ± 3.5 at the base and 14.3 ± 4.9 at the apex (Figures 5A, 6). Neomycin- and artificial perilymph (no vector control)-treated animals showed loss of a significant portion of neurons seen as empty spaces with an average neuronal density of 4.6 ± 0.5 at the base and 5.6 ± 2.3 at the apex (Figures 5B, 6). Additionally, there was a redistribution of neurofilament into the cell bodies within a subpopulation of neurons (Figure 5B). Animals treated with neomycin followed by Ad28.lat.bdnf demonstrated good survival of spiral ganglion neurons with an average neuronal density of 12.6 ± 2.8 at the base and 12.6 ± 3.2 at the apex (Figures 5C, 6). The bodies of the spiral ganglion neurons appeared slightly larger than normal controls or Ad28.lat.ntf3-treated animals (Figure 5C vs. 5D, Figure 6). Animals treated with Ad28.lat.ntf3 demonstrated robust survival of ganglion cells after neomycin with an average neuronal density of 15.6 ± 4.0 at the base and 16.6 ± 4.1 at the apex (Figure 5D, Figure 6). There was a statistically significant reduction of spiral ganglion neuron density in both the basal and apical turn when comparing normal hearing controls with neomycin-treated animals (*p* = 0.0074) (Figure 6). In the basal turn, both Ad28.lat.bdnf- and Ad28.lat.ntf3-treated animals showed statistically a significantly increased density of spiral ganglion neurons when compared with animals treated with neomycin only (*p* = 0.00498 for Ad28.lat.bdnf and *p* = 0.0058 for Ad28.lat.ntf3). In the apical turn, however, only Ad28.lat.ntf3-treated but not Ad28.lat.bdnf-treated animals demonstrated a statistically significant survival of neurons compared with neomycin-only-treated animals (*p* = 0.0058) (Figure 6).

**FIGURE 7:**
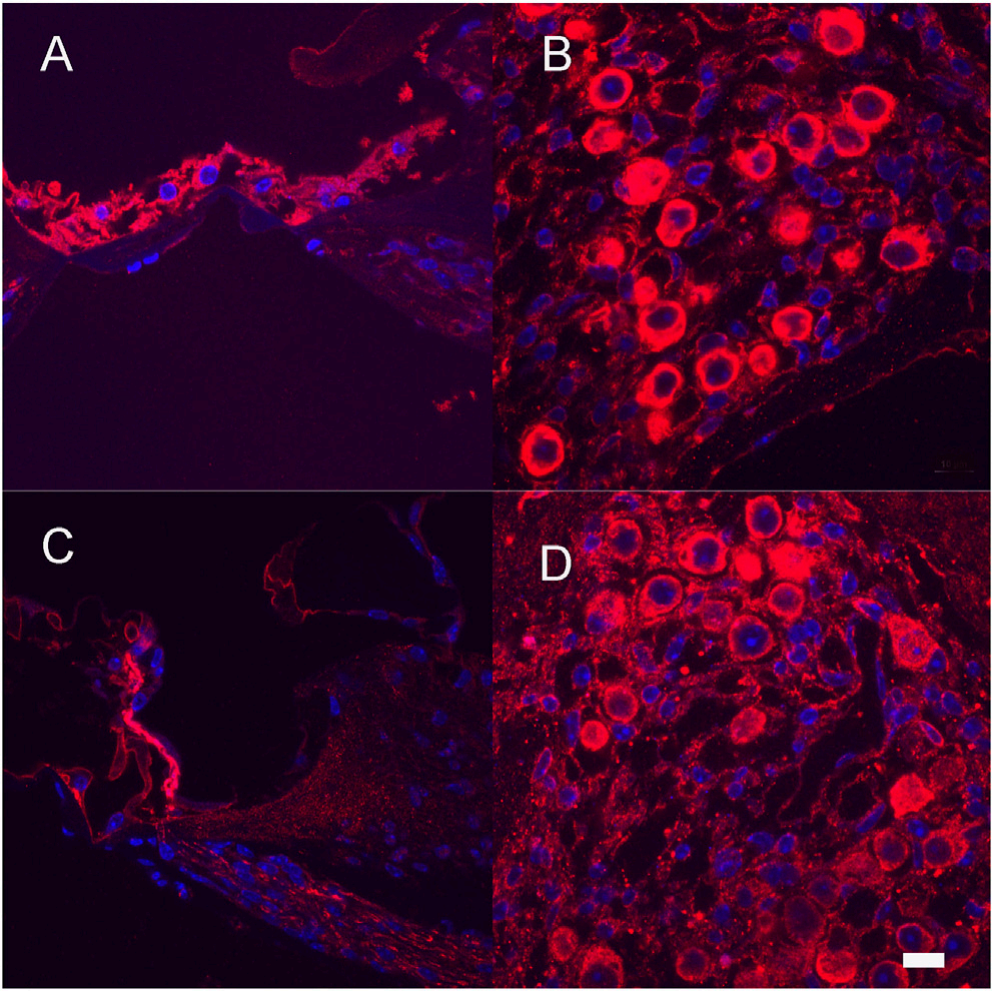
Expression of neurotrophins and their receptors after neomycin treatment and Ad28.lat.bdnf **(A,B)** and Ad28.lat.nf3 **(C,D)**. Expression of BDNF in the damaged organ of Corti **(A)**. Expression of TrkB in the spiral ganglion after neomycin treatment followed by BDNF delivery demonstrating expression of the main receptor for BDNF in surviving spiral ganglion cells **(B)**. Expression of NT-3 in the damaged organ of Corti **(C)**. Expression of TrkC in the spiral ganglion after neomycin treatment **(D)**. Scale bar = 10 µm.

After completion of hearing tests, a subset of vector-treated (Neomycin + Ad28.lat.bdnf or Ad28. lat.ntf3) animals were evaluated to determine the distribution of BDNF and NT-3 and their respective receptors TrkB and TrkC. Expression of BDNF and NT-3 can be seen in the damaged organ of Corti (Figures 7A,C) demonstrating effective delivery of the BDNF and NTF3 genes to the damaged organ of Corti. There is intense immunolabelling for the BDNF receptor TrkB (Figure 7B) and the Ntf3 receptor TrkC (Figure 7D) in the surviving spiral ganglion neurons.

## Discussion

Despite significant advances in signal processing and electrode design, performance of cochlear implants continue to be variable, some of which can be attributed to spiral ganglion dysfunction (Wilson, 2017). A wide variety of growth factors in the inner ear can potentially improve the performance of cochlear implants based on animal models of cochlear implantation. The effect of growth factor infusion into the deaf inner ear can include enhancing the survival of neurons as well as all enhancing and altering the capacity for depolarization of the neurons (Wright et al., 2016). Translation of these observations into successful human studies will require understanding the pathophysiology of neuronal degeneration in human spiral ganglion cells as well as developing systems that restore physiologically relevant levels of growth factors to the inner ear. Potentially, there are differences between human and mouse neurons which could be evaluated through production of human spiral ganglion neurons from iPSCs and *in vitro* comparison to mouse spiral ganglion cells. At present we have only limited data on neurotrophin expression and function in the human inner ear but it does appear that both BDNF and NT-3 are present (Johnson Chacko et al., 2017; Liu, et al., 2011; Shew et al., 2021). In the current study, we demonstrate using a vector system with supporting cell tropism combined with a low-strength long expressing promoter system (herpes latency promoter) is sufficient to maintain survival of spiral ganglion neurons after loss of hair cells and does not induce hearing loss in normal hearing animals.

Long-term delivery of the growth factor(s) using gene therapy has been demonstrated in both cat and guinea pig models of cochlear implantation (Chikar et al., 2008; Leake et al., 2019; Leake et al., 2013; Pfingst et al., 2017). A potential complication of neurotrophin gene therapy is off target effects from transduction of incorrect target cells or the uncontrolled production of growth factor. Recent studies demonstrate the potential side effects of overexpression of growth factors in the normal hearing ear when using gene therapy (Akil et al., 2019; Lee et al., 2016). Similar to Lee et al., we demonstrated that driving NTF3 with the CMV promoter in normal hearing animals resulted in hearing loss and abnormal spiral ganglion neurite growth (Figures 4B,C) (Lee et al., 2016). Similarly, if a vector that overexpresses growth factor was deployed in an ear with residual hearing during cochlear implantation or if the vector is very unevenly distributed within the inner ear after delivery it could potentially induce overproduction of neurotrophins and spread of excess neurotrophins into the perilymph resulting in hearing loss. At present we do not have a direct measurement of neurotrophin expression levels in perilymph but could assume that cerebrospinal fluid may have similar neurotrophin concentrations (de Vries et al., 2019). Concentrations of BDNF have been measured in human cerebrospinal fluid and are present at an average concentration of 1.6 pg/ml (Laske et al., 2007). Our *in vitro* studies suggest that the use of CMV promoters would result in much higher levels of neurotrophin production (Figure 1).

We previously demonstrated that different promoters can produce different effects in terms of the gene expression kinetics and the biological effect induced by different genes in the inner ear (Praetorius et al., 2010). The lat promoter has been widely studied and has been used to drive long-term gene expression (Chattopadhyay et al., 2005). This promoter is a relatively weak promoter that maintains the herpes virus genome inside a cell. As shown in our *in vitro* studies (Figure 1), the use of the lat promoter results in about 80% lower level of neurotrophin production compared with using the CMV promoter. Delivering lower levels of neurotrophins to the inner ear in normal hearing animals resulted in no threshold shift when compared with the delivery of CMV-driven (higher dose) neurotrophins (Figure 4) while still providing effective spiral ganglion support after trauma (Figures 5, 6). This suggests that the use of a low strength promoter system may have a higher safety profile when considering translation to human studies. Ultimately, key to understanding the observed toxicity effects is not only the absolute level of neurotrophin production but also the neurotrophin gradients produced that result in alterations of neuritic growth.

The current studies are limited to only testing two neurotrophins, BDNF and NT-3. Damage to the inner ear results in changes in regulation of a broad range of growth factors (Table 2) (Bailey & Green, 2014; Lefebvre et al., 1994; Wissel et al., 2006). Bailey and Green demonstrated that many of these growth factors have different expression levels with age and also demonstrate basal to apical gradients (Bailey & Green, 2014). The neurotrophins BDNF and NTF3 as well as GDNF and CNTF show a significant acute reduction in gene expression compared with normal controls 1 week after neomycin therapy (Table 2). At 1 month post neomycin treatment, BDNF, NTF3, and CNTF mRNAs are expressed at reduced levels compared with normal hearing controls but at higher levels than in the acute phase of ototoxicity. Neuronal counts in ototoxin-only-treated animals at that time show an approximately 70% reduction in spiral ganglion density (Figure 6), suggesting that the reduced expression of growth factors is not adequate to support spiral ganglion cell survival. A potential explanation for this is that there are disturbances in autocrine signaling within the spiral ganglion or that supporting cell signaling, which is needed to maintain synaptic integrity, is lost after a severe ototoxic trauma (Gomez-Casati et al., 2010; M; Sugawara et al., 2005). Evaluation of mouse models with isolated hair cell loss can be used to demonstrate a much slower progression of spiral ganglion degeneration, potentially allowing us to model human degeneration. These studies, however, do not come to any conclusions regarding the functionality of the nerve as it applies for cochlear implantation. Since electrical stimulation/depolarization alone can induce trophic support of the spiral ganglion, future studies will need to incorporate this into long-term evaluation of growth factor efficacy (Hegarty et al., 1997; Hansen et al., 2001).

There is an additional downregulation of the mRNAs for TrkB and TrkC, the receptors for BDNF and NTF3 that is seen even in the acute ototoxicity period before there is degeneration of the neurons (Table 2). Expression of neurotrophins is known to regulate their receptors, so potentially less production in the organ of Corti results in a change in expression of their receptor mRNAs (Ferrer et al., 1998; Wyatt et al., 1999). It has to be considered that the endogenous BDNF is provided in its pro form and that delivering high amounts of mature BDNF might disturb neurotrophin homeostasis (Schulze et al., 2020). In addition, expression of the corresponding receptors could also be negatively affected. After delivery of low amounts of BDNF or NT-3 to the damaged inner ear with the lat promoter, expression of TrkB and TrkC can be demonstrated in the spiral ganglion (Figures 7B,D). Thus, electrically induced growth factor production and spiral ganglion integrity may be maintained by implanting an inner ear that is undergoing slow degeneration. Human studies do suggest that long-term performance of cochlear implants is stable. For effective translation of a growth factor therapeutic intervention in humans, a predictive test that identifies poor performers or those that could benefit from supplementation of neurotrophins with electrical stimulation needs to be developed.

Further refinements in delivery of neurotrophins can potentially be achieved by using advanced generation vectors. Adenoviral vector systems have traditionally been applied when only short expression of a transgene is needed. In the inner ear, most of these studies are based on Ad5-derived vectors. Causes of inactivation of a vector can include the presence of antibodies against the vector as well as methylation of viral vector DNA. Ad28 vectors are based on a rare serotype human adenovirus that does not have the presence of significant serum antibodies in the human population. Previous studies have shown that this vector targets supporting cells (Hinrich Staecker et al., 2014). In the current study, expression of GFP induced by Ad28 vector could be observed in the residual damaged organ of Corti (Figure 3) and expression of BDNF and NTF3 delivered by this vector could be localized to the damaged organ of Corti (Figures 7A,C). Other rare adenovirus serotype vectors that have less immune reactivity have been used in the eye and demonstrate much longer expression of a vector transgene (Hamilton et al., 2008). An additional advantage of using an adenovector-based system is its significantly larger capacity compared with AAV. Since both NT-3 and BDNF are present in the adult cochlea and probably play different roles, accurate recapitulation of the normal neurotrophin signaling may require delivery of more than one growth factor which can be achieved with a larger vector. Future studies will have to address this and the longevity of gene expression induced by the combination of an Ad28 vector with a herpes latency promoter system.

## Conclusion

Supplementation of neurotrophic factors after auditory injury can be improved by using novel vector constructs combined with targeted vectors. Delivery of BDNF or NTF3 using a rare serotype Ad vector in combination with the herpes latency promoter results in low-level production of neurotrophins that prevents spiral ganglion degeneration after hair cell loss and does not induce hearing loss in normal hearing animals.

## Data Availability

The original contributions presented in the study are included in the article/Supplementary Material; further inquiries can be directed to the corresponding author.
